# HIV self-testing as part of a differentiated HIV testing approach: exploring urban and rural adult experiences from KwaZulu-Natal, South Africa using a cross-over study design

**DOI:** 10.1186/s12889-018-6366-9

**Published:** 2019-01-11

**Authors:** Charlene Harichund, Quarraisha Abdool Karim, Pinky Kunene, Sinenhlanhla Simelane, Mosa Moshabela

**Affiliations:** 1 Centre for the AIDS Programme of Research in South Africa, Durban, South Africa; 2 Department of Epidemiology, Columbia University, New York, USA; 3 School of Nursing and Public Health, University of KwaZulu-Natal, Durban, South Africa; 4 Africa Health Research Institute, Durban, KwaZulu-Natal South Africa

**Keywords:** HIV self-testing, HIV testing, HCT, 90–90-90, Complementarity of HIVST, Unsupervised HIVST

## Abstract

**Background:**

Suboptimal HIV testing rates through available testing approaches such as HIV counselling and testing have directed research efforts toward recognizing the potential of HIV self-testing as an additional testing method. However, HIV self-testing is not readily available within HIV testing facilities and data on how HIV self-testing and HIV counselling and testing will co-exist within HIV testing facilities is limited. Therefore, this study sought to fill this knowledge gap.

**Methods:**

Forty consenting adults were exposed to HIV counselling and testing and HIV self-testing using a cross-over study design between February 2016 and February 2017 resulting in 80 (20,20) interviews. Participants were randomly exposed to HIV counselling and testing first, followed by self-testing, or HIV self-testing first, followed by counselling and testing. In-depth interviews were conducted at baseline, and after each testing exposure, using a semi-structured interview guide. Interviews were transcribed and translated prior to doing the framework analysis.

**Results:**

Support through counselling played a central role in the HIV testing process for some participants who desired support or were not confident to perform unsupervised HIV self-testing. The complementary relationship between HIV self-testing and HIV counselling and testing requires a combination of benefits such as availability of counselling, confidence, convenience and confidentiality (4 Cs) derived from HIV self-testing and HIV counselling and testing. Implementation of the 4 Cs will depend on the availability of unsupervised HIV self-testing and/or supervised self-testing with support from HIV counselling and testing.

**Conclusions:**

As treatment and prevention efforts expand, the reasons for and frequency of testing is changing and there is a need to develop differentiated models for providing HIV testing services to meet client’s needs. HIV self-testing is an important addition to enhance HIV testing efforts and should be offered in combination with HCT.

## Background

The World Health Organization (WHO) recommends the use of HIV self-testing (HIVST) as an additional approach to HIV testing services [1] to enhance HIV testing rates [2]. Stigma, clinic hours, long waiting times at health care facilities and perceived lack of confidentiality associated with HIV counselling and testing (HCT), impede its uptake [3, 4]. The potential of HIVST to overcome the main barriers of HCT has been reported in several studies [5–7]. Additionally, HIVST may enhance health system efficiency as a screening tool by directing human resources toward people with a positive self-test result who are in need of further testing, support and referral, thereby directing services more appropriately [8]. Furthermore, HIVST may be more convenient for users as it displays the potential to reduce the number of facility visits for frequent testers and eliminate the need for individuals to travel long distances or wait in long lines to access HIV testing [9].

Despite the beneficial nature of HIVST in overcoming current drawbacks of HCT, the absence of counselling and accuracy when testing have been highlighted as potential challenges with unsupervised HIVST [10]. Over the years, several HIV testing approaches such as HCT and provider initiated counselling and testing have been added to the HIV testing framework, but have not provided the desired testing uptake due to its dependence of support from health care professionals [11]. While HIVST may require a level of initial supervised support, over time it has the potential to be conducted unsupervised by most individuals [12].

According to recent national HIV testing guidelines, in South Africa, HIVST should be viewed as an additional approach to existing testing services and its use is not intended to displace confirmatory facility-based HIV tests [8]. Public sector health care facilities are the primary access points for HCT in South Africa, with testing services available at no cost. Therefore, to maximize the benefit of HIVST and draw on its potential to improve the uptake of HIV testing, it is important to consider how this modality may be introduced and will co-exist with HCT in primary health care facilities within the public sector. HIVST and HCT may complement each other by one method, overcoming the barriers of the other, but evidence to support their complementarity when both testing methods become available is limited. Thus, the purpose of this study was to explore the validity of notions of complementarity between HCT and HIVST for testing naïve and experienced testers among a population of adults in KwaZulu-Natal, South Africa. Also, to explore how HIVST can be introduced within the HIV testing framework with HCT.

## Methods

### Study setting, design and characteristics of participants

This study was conducted at an urban and rural Centre for the AIDS Programme of Research in South Africa (CAPRISA) site in KwaZulu-Natal, South Africa, which have some of the highest prevalence and incidence rates of HIV [13]. These study sites were selected based on their proximity to primary health care facilities offering HIV testing services, they are centrally located and have access to diverse high-risk urban and rural study populations.

**Fig. 1:**
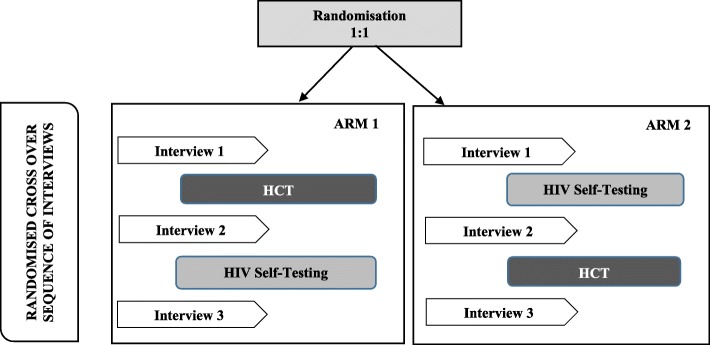
Overview of randomised cross-over study design

The following study design was adopted to explore complementarity between HCT and HIVST and how HIVST can be introduced within the HIV testing framework. Obtaining informed consent from all participants preceded the determination of eligibility in this randomised cross-over designed study. Randomisation envelopes were developed electronically using a 1:1 ratio for participants to receive either standard HCT or HIVST. Once a participant was deemed eligible the research assistant assigned a randomisation envelope in sequential order to the participant. Demographic data was collected for all participants and each participant underwent three in-depth interviews (IDI) on the same day by trained research assistants. The first interview was conducted before the first HIV test, regardless of the assigned study arm; the second after the first HIV test (Fig. 1). Thereafter, those who were assigned to the HCT undertook a self-test, and those initially assigned to self-testing were assigned to HCT (Fig. 1). The third in-depth interview took place immediately after the completion of the second HIV test (Fig. 1). For those in the HIVST group, each participant was provided with a commercially available blood-based HIVST kit (eGenie HIV self-test kits) that included written and graphical instructions on how to complete the HIV test and were allocated a private space to conduct the self-test. For those in the HCT group, HIV counselling was provided by a counsellor who also performed the HIV test as per the standardised HIV testing guidelines of the South African National Department of Health [14]. All participants who tested positive for HIV were referred to the treatment service provided at no cost at the primary health care clinics. The study commenced in February 2016 and concluded in February 2017.

Through purposive sampling, men and women who were either repeat testers for HIV, defined as those who test at HIV testing facilities and have had at least one HIV test in the last year, or were HIV testing naïve were recruited from HIV testing facilities adjacent to the CAPRISA clinical research sites. Participants recruited from the CAPRISA clinic were enrolled in a research study with previous experience(s) of testing (research testers). Eligibility criteria for this study included being HIV uninfected (per participant’s self-report) of unknown HIV status, men and women, > 18 years of age and willing to provide informed consent.

### Data collection

The researchers followed interview guides which were developed based on a scoping review of acceptability of HIVST [15]. Baseline perceptions of HIV testing, HCT and HIVST were discussed during interview 1, while interview 2 was focused on experience with a single testing approach, and interview 3 discussions revolved around experiences with both testing approaches (Fig. 1). Interviews were conducted in either English or isiZulu and each interview lasted approximately 90 min.

### Data analysis

The framework analysis method described by Rabiee [16] was used for data analysis, which included familiarisation of data, identification of a thematic framework, indexing, charting and mapping, as well as interpretation of data. For this study, only data from interviews 2 and 3 were analysed (Fig. 1) as it provided evidence following experience with both testing approaches, and interview 1 was excluded as outcomes were based on perceptions and not experiences as required for this study.

## Results

### Study participants and their baseline characteristics

A total of 40 participants were enrolled in the study, resulting in 80 in-depth interviews following the participants’ exposure to HCT and HIVST. Cohorts were stratified into HIV testing naïve and regular testers as there were no distinguishable differences in reported experiences between repeat testers and research testers. We were unable to recruit male research testers due to limited HIV prevention studies recruiting men within our recruitment areas (Table 1). Also, a higher number of HIV testing naïve women were enrolled, which may be due to more women accessing sexual reproductive health services at the HIV testing facilities. The majority of participants were single, unemployed and completed secondary level education. The average age of men and women were 25 years and 27 years, respectively.

**Table 1:** Demographic characteristics of in-depth interview participants

Demographics	Gender	
	Male	Female
Population (total)	12	28
– Repeat testers	9	9
– Research testers	0	12
– HIV testing naïve	3	7
Average age (range)	25 (19–37)	27 (18–48)
Marital status:		
– Single	11	27
– Married	1	1
Employment status:		
– Employed	3	7
– Unemployed	9	21
Education:		
– Attended university/college	2	9
– Secondary school completed	7	14
– Secondary school not completed	3	5
Access to HIV testing facility		
– Yes	12	28
– No	0	0

### Drawbacks associated with HIV counselling and testing and self-testing during the testing process that may influence complementarity

We used a cross-over study design to eliminate the potential for bias between novel and familiar HIV testing practices among regular and research testers. Through longitudinal analysis we found that as individual testing approaches, optimal use of HCT and HIVST is limited by several drawbacks. However, individually, HCT and HIVST display beneficial characteristics that complement each other and may allow them to co-exist within the HIV testing framework. The main factors associated with HCT and HIVST that influence complementarity are counselling, convenience, confidence and confidentiality.

### Counselling as the backbone of the HIV testing process

Although regular testers were previously exposed to HIV counselling, a need for counselling was still evident during HIVST to prepare a person for testing in the event of a positive result (Table 2) which could be pre-empted by undisclosed risk exposure or assurance that they are not alone during the testing process. Some HIV testing naïve participants exposed to HIVST as their first test reported that absence of counselling denied them support from counsellors (Table 2) that they would have otherwise received during HCT and maintained a need for counselling during the testing process even after exposure to HCT and HIVST. Therefore, HIV counselling may still be required during the HIV testing process and should be made available during the HIVST process for individuals who require it. However, counselling was not universally required among some HIV naïve testers and regular testers.

**Table 2:** Drawbacks associated with HIV counselling and testing and self-testing

Theme	Quotation
Counselling as the back bone of the HIV testing process	*“It’s just that I had not received any counselling I wasn’t ready, so I don’t know how it would have gone had my results been positive.”* (IDI, Male, RT, 0008). *“If you do self-testing, you would not get the support that you get from the counsellor.”* (IDI, Male, HTN, 0033). *“Before you use this kit, you need counselling, if maybe we are going to collect the kit from the clinic, the person who distributes the kit should counsel the user because if I found out that I am positive, alone and I don’t get counselling I might do stupid things.”* (IDI, Male, RT, 0014)
Confidence associated with HIV testing process	*“There would be doubt in the results since you do it yourself.”* (IDI, Female, RT, 0005). *“With HCT, everything will go accordingly in the correct order.”* (IDI, Female, RT, 0020). *“I initially struggled to prick my finger because I was scared that I am going to hurt myself.”* (IDI, Female, RT, 0027). *“It was quiet easy and is a process I can get used to over time, I see HIVST as being quite all right.”* (IDI, Male, RT, 0008)
Convenience during the HIV testing process	*“If you don’t have time to go to the clinic because you work during the week. Some clinics don’t open on weekends, so at least if you buy this kit you can use it in your spare time”* (IDI, Female, HTN, 0031). *“Not being worried about having to go to the clinic to test if you have not been in a while, you can save on transport money.”* (IDI, Female, RT, 0002)
Influence of confidentiality during HIV testing process	*“This (HIVST) can give you privacy and you can decide when and who you want to disclose to” (IDI, Female, RT, 0029). “You don’t have to worry about your results being discussed to other people that you don’t want them to know. Other benefits would be that you do this test at the comfort of your own private space.” (IDI, Female, RT, 0030)*

Note: *RT* Routine tester, *HTN* HIV testing naive

### Confidence associated with the HIV testing process

Some participants believed that testing by trained professionals through HCT was more accurate and reliable (Table 2). Despite regular testers being familiar with the testing process, some were confident to test alone while others were not. By nature of regularly testing, regular testers were not necessarily afraid to test; thus, the fear being referenced could be anxiety associated with a novel procedure which is expected and not related to confidence with self-testing (Table 2). Familiarity with the testing process was not thought to adequately prepare some participants for testing; however, others believed that over time they would develop confidence to do the self-test. Most HIV naïve testers who were exposed to HIVST first, were confident in self-testing and maintained this view even after exposure to HCT.

### Convenience during the HIV testing process

The majority of regular and naïve testers believed that HIVST would offer a convenient testing opportunity for those who either did not have time to present at HIV testing facilities to access HCT or could not afford transport costs or both (Table 2). In our resource limited setting, participants often encounter competing interests between health care and employment, which often leads them to choose employment over health care. Therefore, convenient testing approaches such as HIVST would be desirable.

### Influence of confidentiality during the HIV testing process

Participants were concerned with lack of privacy and disclosure of their results to staff within the health care facilities which may result in exposure to stigma and discrimination (Table 2). Through HIVST, the participants were reassured that testing could be done in a private setting and confidentiality of their results would be maintained, which might influence HIVST as their testing choice.

### Implementation of the 4 Cs model using supervised and unsupervised HIV self-testing

**Fig. 2:**
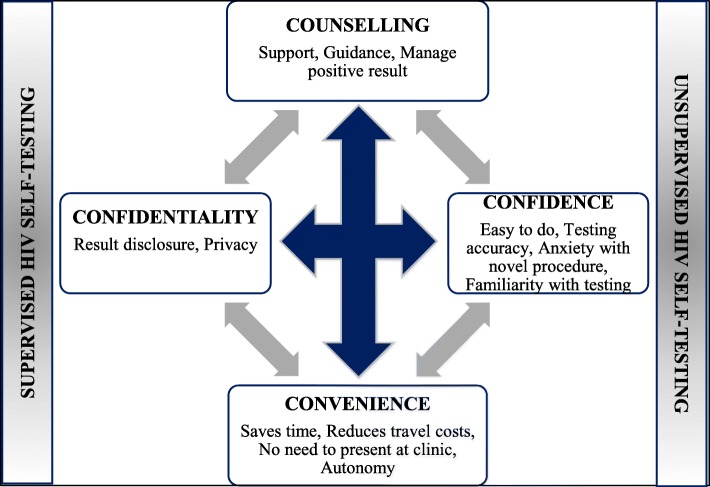
Factors that influence the complementary relationship between HCT and HIVST

The interrelationship of counselling, confidentiality, convenience and confidence associated with HIVST and HCT has led to the design of a HIV testing strategy to support the complementarity of HIVST and HCT, as well as their ability to potentially co-exist within a primary health care setting (Fig. 2).

We found that some HIV testing naïve participants felt confident to perform unsupervised HIVST, but reported that they would have been more comfortable if they had support during the testing process (Table 3). Therefore, these participants might benefit from counselling through HCT or supervised HIVST as their first point of testing and, when comfortable, test through unsupervised HIVST for repeat testing to accommodate their desire for support during the HIV testing process. Despite the definite need for counselling during HIVST as highlighted by most participants, some believed that they would be able to test without counselling and would therefore benefit from unsupervised HIVST.

**Table 3:** Factors affecting supervised and unsupervised HIV self-testing

Theme	Quotation
Confidence and Counselling	*“I can say I was able to use it because I was interested and I wanted to do it and I followed instruction, but if there was someone who was here with me to guide me on how to use it, I would have been more at ease.”* (IDI, Male, HTN, 0006)
Confidence and Counselling in the event of HIV positive result	*“I prefer HIVST because there are instructions to follow to do the test, but at the same time if I discover I am positive, I may not know what to do and will have no one to give me counselling, whereas if there is a counsellor, she would be able to tell me what to do.”* (IDI, Male, RT, 0013)
Confidence, Confidentiality, Counselling	*“HIVST is private and not hard to do by yourself, it’s just the counselling is not there.”* (IDI, Female, RT, 0029)

Note: *RT* Routine tester, *HTN* HIV testing naive

While self-confidence and use of self-testing instructions to perform unsupervised HIVST was evident, the absence of counselling in the event of a positive HIV result was raised as a concern by some participants who lacked confidence to manage a positive HIV result (Table 3). Therefore, counselling from HCT may assist participants who regularly test and still feel that support and guidance from a health care professional is required.

Some participants felt confident to perform unsupervised HIVST and found associated confidentiality with HIVST beneficial to testing, but desired counselling to complete the HIV testing process. In these instances, counselling could again be offered through HCT or supervised HIVST in an attempt to support testing with HIVST. To invoke confidence in participants exposed to HIVST for the first time, supervised HIVST could be introduced. Once participants feel comfortable to perform HIVST independently, they can have a follow-up, unsupervised HIV test.

## Discussion

The complementary relationship between HCT and HIVST suggests a place for differentiated testing options for HIV testing to provide a variety of options for individuals that would accommodate their testing needs that include the 4 Cs – counselling, confidence, convenience and confidentiality – together with options of supervised and unsupervised HIVST. To our knowledge, our study provided a novel strategy (4 Cs) toward introducing HIVST within the HIV testing framework and built on the WHO recommendation of introducing HIVST as an additional HIV testing approach [8]. The 4 Cs indicate the need for flexibility in the HIV testing model to meet the diversity of the needs of clients, rather than a single approach for everyone or clients changing testing needs. According to the WHO, HIVST is a testing approach that could be included in the differentiated HIV testing service delivery model which can be used within and outside health care facilities [17]. In Ghana, HIVST is being introduced through a range of strategies such as supervised, semi-supervised and unsupervised approaches based on an individual’s testing requirements [18]. Our proposed strategy describing how HIVST and HCT complement each other, is further supported by the building blocks of differentiated HIV testing services – when, who, where and what – which could be used to design a client centred HIV testing approach at an individual level [17].

Counselling plays a crucial role in the HIV testing process by providing support during testing, preparing an individual for a HIV test and management of positive results. This concurs with evidence from other studies within and outside sub-Saharan Africa [19–21]. Absence of in-person counselling was concerning to participants in this study, in the event of a positive HIV result as they may not know how to proceed. Having professional support available was also important among men if there was any possibility of a positive HIV result [22, 23]. Telephone counselling was proposed as an alternative to in-person HIV counselling [24], but may not be suitable for all individuals, for example those who have had recent HIV risk exposure. Supervised or semi-supervised HIVST could be offered within HIV testing facilities by health care professionals, or by lay counsellors or trained peers outside a clinic setting, thereby incorporating the ‘who’ aspect of the differentiated HIV testing service model. Whilst this study demonstrates the need for supervised and/or semi-supervised HIVST, further research that explores the logistical implications of this strategy is required.

Benefits such as convenience and confidentiality, underpin the robustness of HIVST as a complementary HIV testing method to that of HCT, which may have a positive effect on HIV testing rates by overcoming the barriers associated with HCT. Hard-to-reach populations who avoid testing at health care facilities due to long waiting times, lack of privacy and possibility of encountering stigma and discrimination [6] may benefit from HIVST. Accommodating participants’ desire for convenient and confidential testing will require a range of access options that will address individuals with a diverse set of needs [22], but we will need to be cognisant of participants’ additional support requirements. Access to HIVST through primary health care facilities may require supervised HIVST for the pre-test counselling component, followed by unsupervised HIVST away from the testing facility to accommodate convenience and confidential testing. Future research is required to explore the feasibility of this approach to provide convenient and confidential testing, while also providing counselling. Affording an individual the opportunity to test ‘where’ and ‘when’ they prefer may increase their desire for testing.

The strength of introducing a novel technology such as HIVST into primary health care facilities may not be fully appreciated until we are able to integrate HIVST into the current HIV testing model. This study provided insight and evidence for policymakers into the complementarity of HCT and HIVST to guide the introduction of HIVST within the primary healthcare domain. The study has several limitations; firstly, given the select sample the study findings are not generalizable, and secondly, we were unable to explore real-time complementarity as HIVST is not routinely available with HCT. Notwithstanding these limitations, the study does provide useful insights on introducing HIVST into primary health care facilities, as HIVST is not available in the public domain in KwaZulu-Natal, South Africa.

## Conclusion

HIVST is suitable as an additional testing approach and should be piloted in combination with HCT given the associated benefits of counselling. The use of HIVST and HCT as a complementary testing approach will need to be tailored to a person’s need by factoring in individual, population and contextual factors. At an individual level, comfort with HIVST will determine whether a combination of supervised and unsupervised HIVST is required, as well as to what extent characteristic components such as professional support is required. Human resource infrastructure to support the demands of interplay between HIVST and HCT will need to be determined at a population level as this may pose a challenge within resource-strained settings. Further research to evaluate real-time use of the complementarity between HCT and HIVST within public health care facilities is needed.

## Abbreviations

HIVST: HIV self-testing
HCT: HIV counselling and testing
CAPRISA: Centre for the AIDS Programme of research in South Africa
IDI: In-depth interviews
RT: Routine testers
HTN: HIV testing naïve
4Cs: Counselling, confidence, convenience and confidentiality
